# Clinical skills of veterinary students – a cross-sectional study of the self-concept and exposure to skills training in Hannover, Germany

**DOI:** 10.1186/s12917-014-0302-8

**Published:** 2014-12-21

**Authors:** Tanja Rösch, Elisabeth Schaper, Andrea Tipold, Martin R Fischer, Marc Dilly, Jan P Ehlers

**Affiliations:** University of Veterinary Medicine Hannover, Competence Centre for e-Learning, Didactics and Education Research in Veterinary Medicine, Hannover, Germany; University of Veterinary Medicine Hannover, Teaching, Small Animal Clinic, Hannover, Germany; Didactics and Education Research in Medicine, University Hospital, Ludwig Maximilian University, Munich, Germany; University of Veterinary Medicine Hannover, Veterinary Director, Clinical Skills Lab, Hannover, Germany; Witten/Herdecke University, Faculty of Health, Didactics and Education Research in Health Science, Herdecke, Germany

**Keywords:** Clinical skills lab, Clinical skills, Questionnaire, Cross-sectional study, Veterinary teaching

## Abstract

**Background:**

Students of veterinary medicine should achieve basic professional competences required to practise their profession. A main focus of veterinary education is on developing clinical skills.

The present study used the guidelines of the “Day-One Skills” list of European Association of Establishments for Veterinary Education (EAEVE) to create an online questionnaire for assessing the skills acquired by students at the University of Veterinary Medicine Hannover (TiHo). The theoretical and practical veterinary knowledge levels of the students and postgraduates are determined and compared.

**Results:**

In two batches, 607 people responded (response batch 1, 23.78%; response batch 2, 23.83%). From 49 defined skills, 28 are actually practised during training at the university and 21 activities are known only theoretically. Furthermore, the students showed great willingness to use simulators and models in a clinical skills lab.

**Conclusions:**

The results of this survey highlight that the opening of a clinical skills lab at the University of Veterinary Medicine Hannover and its incorporation into the study programme are ideal tools to promote practical competences and foster the motivation to learn.

**Electronic supplementary material:**

The online version of this article (doi:10.1186/s12917-014-0302-8) contains supplementary material, which is available to authorized users.

## Background

The acquisition of competences is a fundamental objective in the study of veterinary medicine and an essential component of the Bologna process. Competence-oriented teaching, learning and testing are important aspects of this reform [1].

During their education students should attain all competences necessary for their future careers. Competences form the basis to formulate educational standards and constitute the important learnable cognitive capabilities and skills [2]. Essential skills comprise not only theoretical knowledge and professional attitude, but also acquisition of clinical skills [3].

Clinical skills labs can transmit clinical skills in a realistic environment. Learning on simulation models combines theory and practice. Previously acquired knowledge can be exercised in a hands-on course. The students are able to deepen and retain their knowledge [4].

Clinical skills labs provide innovative learning and testing methods and are implemented almost everywhere in human medicine. As early as the 1970s, medical universities in Maastricht (Netherlands [5]) and in Illinois (the United States of America [6]) introduced clinical skills labs. Since that time, clinical skills labs have been increasingly prevalent, both in human and in veterinary medicine, nationally and internationally. Of the 37 medical schools in Germany, 34 have a clinical skills lab [7]. This demonstrates the importance and high acceptance of these facilities. By the use of clinical skills labs, it is possible to include simulation in medical education, and to “establish it as a teaching platform between theory and bedside” [8].

Internationally, some veterinary faculties have established clinical skills lab concepts already [9,10]. At present in Germany the University of Veterinary Medicine Hannover has an independent, central Clinical Skills Lab (CSL) [3].

At the University of Veterinary Medicine Hannover Students of all semesters can use the CSL both with supervision and on their own. Clinical skills can be introduced to the students already at the first semester to foster motivation and to maintain it over the whole study period. Especially for gaining clinical skills, opportunities to practise repeatedly are indispensable. For ethical reasons, repeated exercises on living animals are often not possible [11]. The clinical skills lab therefore offers students the opportunity for intensive practical training, while maintaining an ethical stance regarding treatment of animals.

The European Association of Establishments for Veterinary Education (EAEVE) defined in its “Day-One Skills” list clinical skills that graduates of veterinary medicine at day one after finishing their studies should know and should be able to perform [12]. This survey documents the skill acquisition of students as compared to these guidelines.

Evaluation and analysis of results examines the following hypotheses:

1. At the start, students possess very little knowledge about veterinary skills, but increase their knowledge during university’s education.
2. The graduates are able to perform essential practical skills and consider themselves as self-competent.
3. The students at the University of Veterinary Medicine Hannover want more hands-on experience. A clinical skills lab can facilitate more practical training.

## Methods

Before opening the clinical skills lab a questionnaire about clinical skills was developed to determine and compare the theoretical and practical veterinary knowledge levels of the students and doctoral candidates.

Based on the EAEVE “Day-One Skills” 49 clinical skills were defined. They are summarized under the following topics (for the exact wording, see Additional file 1 to SOP, EAEVE):

- General clinical skills
- Emergency treatment
- First-aid measures
- Nutritional status
- Laboratory
- Diagnosis
- Animal diseases
- Certificates
- Treatment
- Surgery, anaesthesia
- Euthanasia
- Section, ante-mortem inspection
- Contamination

An online questionnaire consisting of queries about personal data (gender, age and semester, Table 1), and skills-related questions was created.

**Table 1:** **Personal data (n = 607)**

	**Distribution**	**Gender**	**Age**
First Batch		87.8% female	18-47 years
2nd Semester	n=89	12.2% male	
4th Semester	n=84		Av. 25.08 years
6th Semester	n=94		
8th Semester	n=66		
10th Semester	n=82		
Completed degree	n=131		
Second Batch	n=61	86.7% female	18-32 years
1st Semester		13.3% male	
			Av. 21.25 years

Half a year before opening the clinical skills lab, all students registered for summer semester 2012 at University of Veterinary Medicine Hannover (n = 2296) received a link to the questionnaire via e-mail. For this first batch students of the second to tenth semesters and postgraduates (designated in the questionnaire as “completed degree”) were queried.

The same questionnaire was sent shortly before the beginning of the winter semester 2012/2013 to the second batch of students of the new first semester (n = 256) to assess their state of knowledge prior to beginning their studies.

The questionnaire was open for two months, and the link was sent to the students several times as a reminder. The study was conducted according to the ethical rules of the University. The officer of data protection Prof. Dr. Bernd Schröder and the doctoral committee of the university gave their consent to the proposed project. All data obtained were processed and evaluated anonymously and in accordance with the EU Directive 95/46/EC.

### Question I: Please rate the following clinical skills

Response options:

1. I do not know this skill.
2. I know the theory of this skill.
3. I have seen this skill practised.
4. This skill was shown and explained to me by a veterinarian.
5. I have performed this skill myself under the supervision of a veterinarian.
6. I have performed this skill without supervision.
7. I am able to perform this procedure independently.

The participants should assess their knowledge about the mentioned clinical skills.

Therefor these response options were choose, so that it was possible to make a differentiation between lake of knowledge, knowledge, performance and mastery.

For further evaluation the answers to this question were put into two categories:

- *Category A*: Answers 1–4. Skill unknown or observed in practice
- *Category B*: Answers 5–7. Skill performed personally

Based on this classification this question was evaluated according to the following groupings:

Grouping a) which skills students are able to perform after graduating?

To demonstrate how skills are attained from theoretical knowledge up to independent performance the following classification was made. The skills were assigned to the grouping a) if the answers of students in semesters 1–4 were over 50% in *Category A,* the answers of students in semesters 6–8 were in *Category A* or *B*, and the answers of students in semester 10 and of those with completed degree were over 45% in *Category B*.

With this classification the development from the knowledge to the ability to perform the clinical skill can be expressed. In the preclinical semesters (1^st^ to 4^th^) most of the students (over 50%) did not know the skills or observed it in practice. In semester six to eight the students learned more about the skills and a few of them also performed them on their own. Based on this, the answers of the students at 6^th^ and 8^th^ semester were also in *Category A* or *Category B*, a percentage could not be mentioned. Most of the students of the 10^th^ semester and those with degree (over 45%) had performed the skills personally.

Grouping b) which skills do most students of all semesters know only in theory or by observing?

The skills were assigned to this grouping if over 50% of the answers of all respondents were assigned to *Category A*.

Grouping c) Which skills were marked as unknown by most of the students?

Skills were assigned to this criterion if the majority of answers for each semester were response 1.

### Question II: How did you get to know this skill?

Response options:

1. I do not know it.
2. It was explained to me.
3. I have observed or done it on an animal.
4. I have observed or done it on a simulator.
5. I have observed or done it on an animal and on a simulator.

### Question III: Would you like to practise these skills on a simulator?

Response options: Yes or No

### Question IV: Which simulators and models would you like to have in the clinical skills lab?

Free text answer

Survey data were initially captured using SurveyMonkey®. The personal data and free-text responses were evaluated using the spreadsheet program Microsoft® Office Excel 2010 (Microsoft Corporation, California, USA).

The distributions of characteristics were assessed by the chi-squared homogeneity test procedure FREQ, using the statistics program SAS® (Version 9.3, SAS Institute GmbH, Germany). Because of the textual characteristic of the responses this test was selected to generate percentage distributions.

## Results

At the summer semester of 2012 546 people answered the survey (response rate 23.78%).

The survey among first-year students in the winter semester 2012/2013 gleaned 61 completed questionnaires (response rate 23.83%).

Table 1 shows the evaluation of the personal data.

### Question I

**Figure 1:**
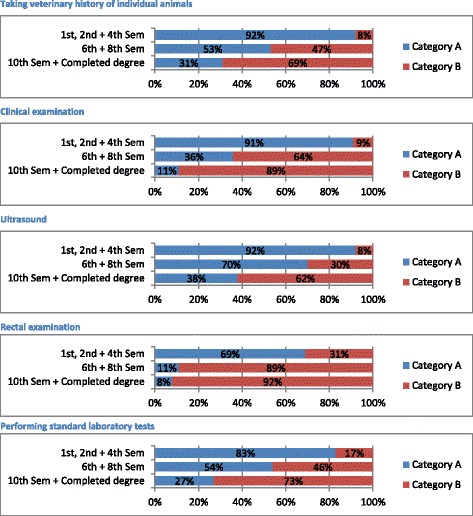
**Five representative skills for grouping a) Question I: “Please rate the following clinical skills”.** Category A: Answers 1–4. Skill unknown or observed in practice. Category B: Answers 5–7. Skill performed personally.

Twenty-eight skills were assigned to grouping a). Five representative skills were shown in Figure 1 See Additional file 2 for the remaining diagrams.

**Figure 2:**
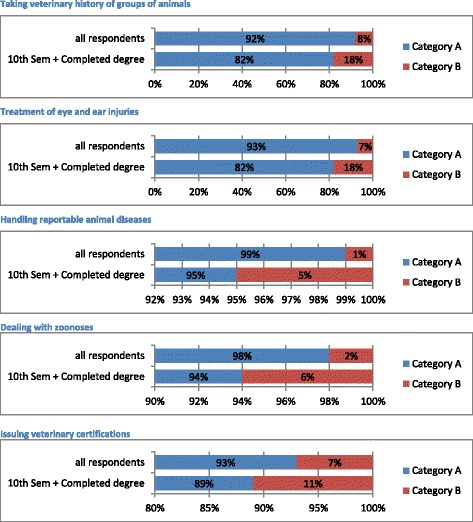
**Five representative skills for grouping b) Question I: “Please rate the following clinical skills”.** Category A: Answers 1–4. Skill unknown or observed in practice. Category B: Answers 5–7. Skill performed personally.

The remaining 21 skills appropriated grouping b). Five representative diagrams are shown in Figure 2, the remaining skills are displayed in Additional file 2.

**Figure 3:**
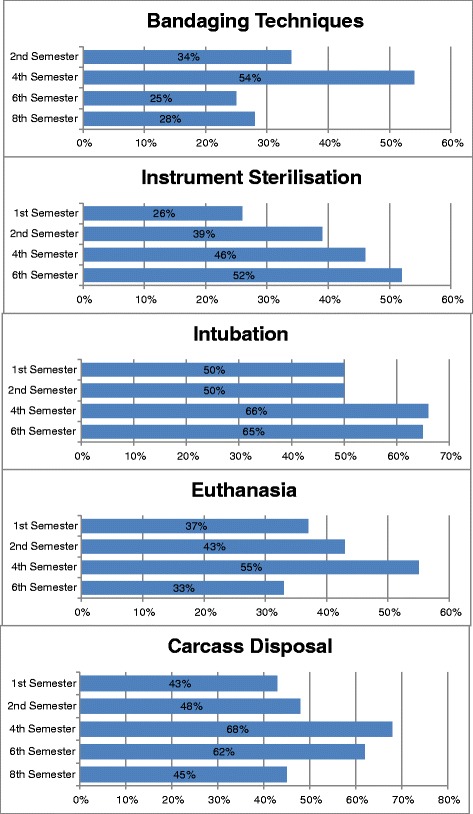
**Five skills for grouping c) Question I: “Please rate the following clinical skills”.** The most students marked these skills as unknown.

Five skills received remarkably high percentages of response choice 1. These skills that most students marked as unknown, determined from Question I were assigned to the grouping c), and are shown in Figure 3

.

### Question II: How did you get to know this skill?

The analysis of this survey showed that at beginning of study most skills are not known by the students. During the study of veterinary medicine the skills are explained, or the students observed the performance or performed it by themselves on an animal.

Few students have already seen or performed obstetrics or skin and intestinal suture exercises on a simulator.

Some students reported seeing or performing the following procedures on an animal or a simulator: restraint; clinical examination; taking, storing, and transporting samples, obstetrics; rectal examination; injection techniques and skin suture practice.

### Question III: Would you like to practice these skills on a simulator?

First semester students indicated that they would like to practice every skill on a simulator.

Higher semester students indicated that they would like to practice most skills on simulators. Opinions were divided on the following skills (yes: no).

- Restraint (54.7%:45.3%)
- Assessment of nutritional status (55%:45%)
- Explanation of treatment (59.1%:40.9%)
- Sterilisation of instruments (58.7%:41.3%)
- Responding to feelings, maintaining security (52.3%:47.7%)
- Carcass disposal (53.6%:46.4%)

### Question IV: Which simulators and models would you like to have in the skills lab?

**Figure 4:**
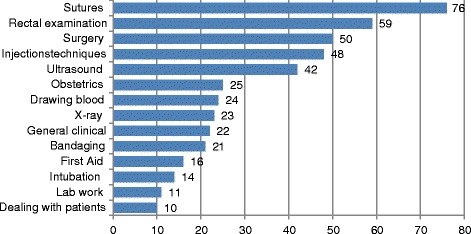
**Desired practice facilities with 10 or more recommendations for Question IV.** Skills (n = recommendations).

In total, 45 skills were named. In Figure 4 skills with ten or more recommendations are listed. Most frequent wishes by the students were suture techniques, rectal examination, surgery and injection techniques.

First semester students wished practice opportunities for those clinical skills that are neglected for their later practical occupation

The students of higher semesters and graduates expressed a desire for surgical exercises to reinforce the lessons in the curriculum.

In addition some respondents (14/ 206) voiced misgivings about the simulators. These students would prefer practise on living animals and were afraid of that the introduction of simulators could displace training on animals.

## Discussion

The aim of this study was to determine the level of knowledge of practical clinical skills of students and postgraduates.

Therefore an online questionnaire was used. It is considered comparable to the traditional survey methods in productiveness and validity [13]. The anonymity of the respondents is a main advantage of a survey on internet. Because of this the respondents are often more honest [14]. Also, in contrast to a personal inquiry, the interviewer cannot influence the respondent.

The return rate of the surveys could be determined because all enrolled students and postgraduates could be reached through their semesters’ batch e-mails. The return rate is similar to that of comparable studies [15] and with about 24% within the range of 5-30% expected for written surveys [16]. The study can be considered representative because of the high number of participants and broad survey of all semester groups.

The 49 skills on the questionnaire were derived from the guidelines of the “Day-One Skills List” of EAEVE. Graduates of veterinary medical studies could master the techniques and can perform the procedures, which are mentioned in the list [12]. In Germany the veterinary licensing regulations (so called TAppV) specify transmission of practical skills for future autonomous, independent professional practice as a goal of veterinary education [17].

The results show that of these 49 skills, 28 are intensely practised during the course of study and are mastered, while 21 are known only in theory or from observation.

Six skills were marked as not known by some respondents up to and including some in the tenth semester (bandaging techniques, issuing veterinary certificates, instrument sterilisation, intubation, euthanasia and carcass disposal). This result is in contradiction to the curriculum and the content of the lectures and exercises. Bandages, sterilisation of surgical instruments, intubation and euthanasia are topics in the curriculum that are either demonstrated or practised in instruction sessions. These topics should be known to most students, at least in theory and through the extramural internships that start after the fifth half-year of study [18].

The purpose of carrying out one study [19] was to find out whether veterinary education meets legal standards. The present results make transparent the “definite discrepancy between what a graduate at the end of an education should by law be able to do, and what he realistically in practice can do” [19]. The cause of the discrepancy has been thought to be the theoretical emphasis in training, the high number of students and the lack of opportunity to transfer theoretical knowledge into practice.

In Germany veterinarians are not satisfied with the practical competence of their assistants and judge that the teaching of practical clinical skills during studies should be improved [19,20]. As occurred with veterinary students, many students from medical schools also rate themselves as insufficiently trained in history taking, clinical examinations and diagnostics [21].

Learning centres like clinical skills labs offer the possibility to counter this situation by offering a place to train practical competences and create practice opportunities within the curriculum.

The study of veterinary medicine is a scientific study providing the basis for practice as a professional veterinarian [22]. During their education, students are trained comprehensively in theoretical knowledge and veterinary skills and prepared for their future profession. Because of the discrepancy of the number of students and the actual number of patients respectively diseases seen in clinics and because of ethical considerations exercises to acquire practical skills using live animals are limited and not accessible to every student. Current graduates of veterinary medicine studies have excellent theoretical knowledge and know about the current practical clinical skills. As the results clearly illustrate, however, there is need for more practice performing the procedures. Changes in the Veterinary Licensing Regulations (from TAppO [23] to TAppV [17]) recognise this, so that veterinary education was expanded to include more practical and competence oriented areas [24]. Besides the necessary knowledge and professional attitude, students should also become familiar with practical skills [25]. It is likely that students and graduates gain some of the skills required in extramural practical works and particularly in their practical year [24], as well as during their beginning employment as assistants.

In focusing on a more practice oriented education, principles of “constructive alignment” [26] should be observed. The curriculum should be designed according to which learning outcomes and professional competences are to be transmitted and which methods are appropriate to test them [27]. Teaching methods must be chosen in a way that the results of the learning and skills acquisition process cover the desired outcomes. Following constructive alignment principles can promote learning of deep knowledge and is effective [28-30]. The tests, as well, should be oriented to these learning outcomes [31]. Because university students are especially susceptible to the “assessment drives learning” effect, they focus their learning process strongly on tests [32].

Teaching and learning in a clinical skills lab make it possible to use the principles of constructive alignment, particularly focusing more on the students and their personal learning strategies.

To make the students aware that the CSL is an important and mandatory part of teaching, experts recommend to use the clinical skills lab also for exams [20]. With the introduction of an objective structured clinical examination (OSCE), practical clinical skills can be validly and effectively tested [33-36]. Moreover, the effectiveness of the skills lab trainings can be investigated and proven with it [37,35].

Interestingly, the results showed that some students in the first semester rank significantly better than students in higher semesters. In one survey [38], similar results were found in a self-assessment of their communication skills by students of veterinary medicine. Another investigation [39] documented that especially those students whose accomplishments are weaker overestimate their skills. By contrast, students with good grades usually assess themselves worse. Generally, veterinary students are considered very confident in their self-assessment of their skills [40].

The survey took place before the beginning of the semester, so that some respondents had little experience about what to expect and what a university education requires, and are highly motivated. In the first year of pre-clinical [41] study of veterinary medicine, there is much theory and less animal contact. Motivation drops because of theoretical preclinical subjects [42].

A skills lab accessible for all semesters gives students the option to gain clinical skills starting in the first semester. This option can maintain the strong motivation present at the start of university, strengthen self-confidence, and improve self-assessment. Such voluntary exercises in addition to the required studies are generally accepted enthusiastically by students. Utilization of the facility and equipment by students from Medical schools is excellent [43-45].

The effectiveness of skills lab training and its high level of acceptance among students have been tested and proven in numerous studies [46-49] and are further supported by the present study.

Simulators are increasingly well received in veterinary medical education, since the number of patients for teaching purposes is limited and the models present standardized repeatable procedures at any hour and as frequently as desired [50-53]. Thus, simulators can contribute to graduating more confident, competent veterinarians [54].

Evaluation showed that the respondents still have very little experience in the use of simulators. One explanation for this is that the survey took place before the opening of the skills lab at the University of Veterinary Medicine Hannover. So far simulators have been used sporadically circumferential facilities. Another reason could be the little amount of commercially available veterinary simulators, in comparison to the availability in human medicine. Such simulators have to be developed and built in veterinary medicine first, as it has previously happened [53].

The willingness and desire to practise and demonstrate practical clinical skills on simulators exist among the students and are very high. In almost all cases, all respondents indicated that they would like to train on models. There were only six skills where opinions were divided. On one hand, the respondents certainly find it more reasonable to learn about coercive measures and assessment of nutritional status with live animals, since here, animal reactions and interactions are particularly important.

On the other hand, the communicative nature of the skills (counselling, talking with patient owners) should be mentioned. Properly simulation patients (SP) were not known [55].

Precisely in communication skills such as bringing bad news or dealing with difficult patient owners, the students feel insufficiently trained and prepared [56]. The ideal setting to learn and practise these skills is in a skills lab with simulated patient owners.

The results on desired models showed that most of the simulators which the students wanted are already present in the skills lab or at least are in planning [57]. In the evaluations, there were some critical remarks about the simulators and the clinical skills lab. Some students would prefer, instead of simulators, more practice on living animals, since they see a danger that the CSL puts training on animals in the background. A survey conducted among students and faculty of the university before the opening of the skills lab found similar results [20].

However, practising on living animals is regarded as an intervention to the animal and is controversial [58]. Increase in animal population and using more live animals are therefore ethically not accepted. Because of animal welfare considerations, alternatives to animal experimentation are increasingly sought for veterinary teaching [59-62]. Use of simulators and the introduction of skills lab training offer the perfect solution. Studies [63-65] demonstrate the complexity of existing models and the ways to use them, so that certainly simulators can replace training on living beings or prepare students well for them.

In summary, the hypotheses formulated earlier can be discussed as follows: At the start of their studies, students possess very little knowledge about veterinary skills, but gain good knowledge during their education for a large proportion of these skills. However, the results also showed that they can acquire not all of the required skills during their studies. Graduates assess their competence within limits. There were very few skills which a majority of students stated that they had mastered. This study showed that the acquisition of practical skills in veterinary medicine could be improved. The new Clinical Skills Lab has great potential and could enhance the quality of education. It not only offers the possibility to create the new practical exercise opportunities for students, but can also thereby help students towards realistic self-assessment.

The data from this inventory provide important information for building up and establishing skills lab concepts in veterinary medicine. Further investigations are needed to clarify the status in other countries and/ or during curriculum development. On the basis of this survey, a longitudinal study could be conducted to advance and adapt the development of clinical skills labs and their integration into veterinary teaching.

## Conclusions

The results of this survey demonstrate that in veterinary education, the transmission of practical skills should be improved and include more communication and practical professional skills.

To implement uniform educational standards, as in human medicine, a competency-based learning target set for practical skills should be developed and the scope of this training reflected in the curriculum. A clinical skills lab provides a good opportunity to develop and establish standardized practical teaching. In addition, it offers the option of practising repeatedly specific skills tailored to personal preferences or the desired career.

Opening a clinical skills lab at University of Veterinary Medicine Hannover and the opportunity to incorporate it into the veterinary education are steps towards fostering practical skills and maintaining the motivation to learn.

## Additional files

Additional file 1:
**List of the requested clinical skills in the exact wording.** This file contains the exact wording of the requested 49 clinical skills, which were used for the study according to the “DAY-ONE SKILLS” list of the EAEVE (European Association of Establishments for Veterinary Education). Additional file 2:
**Remaining diagrams.** This file contains the remaining diagrams from Question I: Please rate the following clinical skills for the grouping a) and grouping b).
