# Supplementary material for: Clinical skills of veterinary students – a cross-sectional study of the self-concept and exposure to skills training in Hannover, Germany
Source: BMC Vet Res. 2014 Dec 21;10:969. doi: 10.1186/s12917-014-0302-8 (PMC4300046; doi:10.1186/s12917-014-0302-8)
Supplement: Additional file 1: — List of the requested clinical skills in the exact wording. This file contains the exact wording of the requested 49 clinical skills, which were used for the study according to the “DAY-ONE SKILLS” list of the EAEVE (European Association of Establishments for Veterinary Education). [file 12917_2014_302_MOESM1_ESM.docx]

**Additional file 1: List of the requested clinical skills in the exact wording**

- General clinical skills

- - Taking veterinary history of individual animals
  - Taking veterinary history of an animal group
  - Handling and restraining an animal
  - Perform a complete clinical examination of an animal

- Emergency treatment

- - First aid management of haemorrhage
  - First aid management of wounds
  - First aid management of breathing difficulties
  - First aid management of eye and ear injuries
  - Treatment of unconsciousness
  - First aid management of burns
  - First aid management of internal organ damage

- First-aid measures

- - Bandaging
  - Treatment of Injures
  - Immobilising limbs
  - Resuscitation procedures

- Nutritional status, Lab work

- - Assess correctly the nutritional status of an animal
  - Advise the client on principles of husbandry and feeding
  - Collect, preserve and transport samples
  - Perform standard laboratory tests
  - Interpret the results of those generated in-house, as well as those generated by other laboratories

- Diagnosis, Animal diseases, Certificates

- - Use radiographic
  - Storage of an animal for radiography
  - Radiation (X-Ray)
  - Use ultrasound
  - Detection of organs in ultrasound diagnostics
  - Follow correct procedures after diagnosing notifiable and reportable diseases
  - Follow correct procedures after diagnosing zoonotic diseases
  - Carry out Certification correctly

- Treatment

- - Advise on, and administer appropriate treatment
  - Access the appropriate sources of data on licensed medicines; prescribe and dispense medicines correctly and responsibly in accordance with relevant legislation
  - Ensure that medicines and waste are safely stored and/or disposed of
  - Obstetrics
  - Rectal examination
  - Injection techniques

- Surgery, anaesthesia

- - Correctly apply principles of sterilisation of surgical equipment
  - Correctly apply principles of aseptic surgery
  - Intubation
  - Suture exercises (skin)
  - Suture exercises (intestinal)
  - Safely perform sedation
  - Safely perform general anaesthesia
  - Safely perform regional anaesthesia
  - assess and control pain

- Euthanasia, Section, ante-mortem inspection, Contamination

- - Recognise when euthanasia is necessary and perform it humanely
  - Show sensitivity to the feelings of owners and others, and regard the safety of those present
  - Advise on disposal of the carcass
  - Perform a basic gross post mortem examination, record details, sample tissues, store and transport them
  - Perform ante and post mortem inspection of food animals and correctly identify conditions affecting the quality and safety of products of animal origin
  - Minimise the risks of contamination, cross infection and accumulation of pathogens in the veterinary premises and in the field
