# Supplementary material for: Clinical skills of veterinary students – a cross-sectional study of the self-concept and exposure to skills training in Hannover, Germany
Source: BMC Vet Res. 2014 Dec 21;10:969. doi: 10.1186/s12917-014-0302-8 (PMC4300046; doi:10.1186/s12917-014-0302-8)
Supplement: Additional file 2: — Remaining diagrams. This file contains the remaining diagrams from Question I: Please rate the following clinical skills for the grouping a) and grouping b). [file 12917_2014_302_MOESM2_ESM.docx]

**Additional file 2: Remaining diagrams**

**Question I: "Please rate the following clinical skills"**

Category A: Answers 1-4. Skill unknown or observed in practice

Category B: Answers 5-7. Skill performed personally

Grouping a) Which skills students are able to perform after graduating?

**Taking veterinary history of individual animals**

**Handling and restraining**

**Clinical examination**

**Bandaging techniques**

**Cleaning wounds and injuries**

**Assessment of nutritional status**

**Housing and feeding advice**

**Sample taking, transport and storage**

**Performing standard laboratory tests**

**X-ray diagnostics**

**Positioning (X-ray)**

**Shielding (X-Ray)**

**Ultrasound**

**Recognition of organs (ultrasound)**

**Explanation of therapy**

**Medication**

**Rectal examination**

**Injection techniques**

**Principles of aseptic surgery**

**Intubation**

**Suture exercises (skin)**

**Sedation**

**General anesthesia**

**Local anesthesia**

**Assessment of pain responses**

**Euthanasia**

**Section**

**Ante-mortem and post-mortem inspection**

Grouping b) Which skills do most students of all semesters know only in theory or by observing?

**Taking veterinary history of groups of animals**

**Emergency treatment bleeding**

**Emergency treatments for wounds**

**Treatment of acute dyspnea**

**Treatment of eye and ear injuries**

**Treatment of unconscious animal**

**Treatment of burns**

**Treatment of internal injuries**

**Immobilization of extremities**

**Resuscitation**

**Interpretation of laboratory results**

**Handling reportable animal diseases**

**Dealing with zoonoses**

**Issuing veterinary certifications**

**Proper storage and disposal of medications**

**Obstetrics**

**Sterilisation of surgical instruments**

**Suture (intestinal)**

**Responding to the feelings of the owner + maintaining safety (euthanasia)**

**Carcass disposal**

**Dealing with contamination and cross-infection**
